# Safety profile of miltefosine in the treatment of cutaneous leishmaniasis

**DOI:** 10.1371/journal.pone.0315710

**Published:** 2024-12-13

**Authors:** Laís Raquel Ribeiro, Sarah Nascimento Silva, Mell Ferreira Saliba, Janaína de Pina Carvalho, Gláucia Cota

**Affiliations:** Pesquisa Clínica e Políticas Públicas em Doenças Infecto-Parasitárias, Instituto René Rachou, Fundação Oswaldo Cruz, Belo Horizonte, Minas Gerais, Brazil; Universidade Federal da Bahia, BRAZIL

## Abstract

Cutaneous leishmaniasis (CL) is a neglected tropical disease that poses a significant public health challenge in Brazil and worldwide. Miltefosine, the only orally administered drug available for CL, was recently incorporated into Brazil’s treatment protocols following recommendations by the World Health Organization (WHO) and revisions by national health authorities. While this represents an important advancement, miltefosine is associated with frequent gastrointestinal side effects and potential teratogenic risks, necessitating careful patient eligibility assessments and close clinical monitoring throughout treatment. Furthermore, the absence of national effectiveness data underscores the need for careful monitoring during large-scale implementation. This study, part of a broader implementation monitoring process, seeks to estimate the frequency, intensity, and seriousness of adverse events (AEs) associated with miltefosine. It also aims to identify factors linked to treatment discontinuation during the pilot phase of miltefosine distribution in the state of Minas Gerais, Brazil. Descriptive analyses were performed to present measures of central tendency and dispersion for the variables. Additionally, a multivariate analysis was conducted to explore relationships between explanatory variables and outcomes of interest. Between 2021 and 2023, 77.1% of patients treated with miltefosine experienced at least one AE. The rate of serious AEs related to treatment was 1.3%. Gastrointestinal symptoms were the most commonly reported AEs, followed by musculoskeletal manifestations. The most frequent laboratory alteration observed was an increase in serum creatinine, which was significantly associated with hypertension, age, and mucosal involvement of leishmaniasis. No pregnancies were recorded during the implementation period. Early treatment discontinuation rate occurred in 11.8% of cases, with discontinuation associated with age and baseline serum creatinine alterations. Half of the patients required temporary treatment interruptions or irregular dosing, extending the treatment duration beyond the planned 28 days. This pharmacovigilance model provides valuable insights, representing an approach potentially applicable to other neglected disease control programs, especially when introducing new treatment technologies.

## Introduction

Cutaneous leishmaniasis (CL) is a globally distributed infectious disease, with over 200,000 cases officially reported in 2022 [1]. It is classified as a neglected tropical disease, and Brazil accounts for 85% of global cases, alongside seven other countries [2]. The disease is caused by various species of *Leishmania*, transmitted through the bite of infected female sandflies. The clinical presentation of CL varies depending on the interaction between the parasite and the host, largely influenced by the patient’s immune response [3]. The typical manifestation of CL in the Americas is a round ulcer with elevated and infiltrated edges, usually appearing on exposed areas of the body, particularly the face and extremities. Other forms of the disease include the disseminated cutaneous form, which presents with lesions spread across the body, and the mucosal form, primarily affecting the upper airways, especially the nose mucosa [4].

Therapeutic options for CL treatment remain limited, with most requiring parenteral administration, prolonged courses, and posing significant toxicity risks. In recent years, new therapies have been explored to expand access, offering more convenient dosing and reduced toxicity, particularly with the development of local treatments [5–8].

Miltefosine is the first oral treatment incorporated into the Brazilian Public Healthcare System for CL, following a lengthy incorporation process [9–11]. The drug is now considered a first-line treatment for CL but requires strict regulation due to its potential teratogenic effects. Its use is closely monitored, necessitating patient liability documentation, the prolonged use of contraceptive methods for women of childbearing age, and ongoing pregnancy monitoring during treatment [12]. To address the various challenges associated with the implementation of this new therapy, a local study was designed to support the introduction of miltefosine in the state of Minas Gerais, in collaboration with the Health Department of the State of Minas Gerais [13].

Some controlled studies have examined the efficacy and safety of miltefosine [14–19]. Real-world studies are crucial for providing safety data on the drug, particularly given its association with teratogenicity and adherence concerns. In this regard, this study aims to contribute real-world evidence to the process of technology incorporation in Brazil, validating its actual benefits and guiding the leishmaniasis control program. Our objective was to evaluate the safety profile of miltefosine during the initial implementation phase for the treatment of CL in Minas Gerais.

## Methods

### Study design

This is an observational, prospective cohort study aiming at reporting safety monitoring during the pilot phase of miltefosine distribution for CL in Minas Gerais.

### Intervention and outcomes

Patients received treatment for CL with miltefosine in 50 mg capsules, administered orally in two or three daily doses for 28 days. The outcomes of interest in this analysis included i) clinical AE rate, ii) laboratory AE rate, iii) overall AE rate, iv) early treatment interruption rate, and v) laboratory AE rate stratified by intensity.

### Participants and eligibility

The study population comprised all patients diagnosed with CL and treated with miltefosine in Minas Gerais from July 2021 to August 2023. This period was chosen as it encompassed the pilot phase of implementing the first 200 treatments in this Brazilian state. The prescription and use of miltefosine are governed by local legislation, which includes specific requirements such as detailed instructions and a set of forms necessary for dispensing the drug. Among these requirements, signing an informed consent form is mandatory. Additionally, patients of childbearing age with the potential for pregnancy were required to use highly effective contraception methods for 30 days before starting miltefosine treatment, throughout treatment, and four months after its completion, following the recommendations of local legislation [20] to combine one highly effective method with one barrier method. To enhance control over the distribution of miltefosine and ensure that medical and laboratory assessments occurred throughout treatment, medication dispensing was organized into two stages, with a 14-day supply provided at each stage. Patients who received at least one dispensing stage of miltefosine and whose monitoring form was available, even if incomplete, were eligible for the safety analysis. Treatment was defined as complete if administered over 90% of the indicated days. Patients without a filled-out safety monitoring form were excluded from the analysis.

### Sources and data collection

A database was created to compile administrative variables related to medication requests, as well as sociodemographic and clinical data of the patients. The data sources included the Notifiable Diseases Information System (SINAN) form (S1 Table), the miltefosine request form (S2 Table), and the safety monitoring form (S3 Table). Physicians responsible for patient care completed all the required forms and reported any AEs observed throughout the miltefosine treatment. The observation period for reporting AEs in this study extended from the initiation of miltefosine treatment until the first medical consultation following the completion of treatment. The treating physicians recorded events based on patient reports obtained through a directed anamnesis aimed at actively identifying occurrences and through the analysis of test results conducted during the monitoring period.

Databases were accessed for research purposes from May 18, 2023, under a confidentiality and secrecy agreement signed with the coordination of pharmacy, therapy, and care of the Health Department of the State of Minas Gerais. The researchers verified the consistency of the data and identified duplicates based on the records of medication request forms and dispensing records. Several data validation mechanisms were implemented, including the exclusion of duplicates, cross-checking information from the monitoring forms with official miltefosine dispensing records, and clarifying any discrepancies or unclear information, including serious adverse events (SAEs), with the physicians via phone calls. At the end of the treatment period, the monitoring form was emailed to the physicians. Additionally, reminders were sent via emails, text messages, and phone calls to prescribers and the healthcare team regarding the importance of monitoring patients on miltefosine [13].

### Study conduct and assessment of adverse events

The variables of interest included the primary demographic and clinical characteristics of the patients, such as gender, age, comorbidities, clinical form of leishmaniasis, duration of lesions, and whether it was the first episode or a recurrence. All AEs that occurred during the administration of the medication were documented. The classification of AEs adopted in this study was based on guidelines from the Brazilian Health Regulatory Agency (ANVISA) [21]. An AE was defined as any signs, symptoms, changes in laboratory tests compared to reference values, worsening of the baseline clinical condition, or any other unwanted medical occurrence, regardless of their causal relationship with the administered product [21].

While the monitoring form included sections for assessing causality, most were left unfilled by the reporters, which made it challenging to apply standard causality assessments. Nonetheless, based on the brief clinical reports provided, it was still possible to classify causality through clinical judgment. Events were categorized as either "related" (including the defined, probable, or possible categories established by WHO) or "not related." This classification considered factors such as temporal relationships, pharmacological and pathophysiological plausibility, and the presence of alternative causes [22, 23].

Regarding seriousness, an AE was classified as an SAE if it resulted in death, posed a threat to life, caused persistent or significant disability/invalidity, necessitated hospital admission or prolonged hospitalization, or led to a congenital anomaly or congenital disability. Any suspicion of transmission of an infectious agent through the medication or any clinically significant event was also considered an SAE [24].

AEs were characterized by their system-organ involvement and allocated to their respective System and Organ Classes (SOC) using the Medical Dictionary for Regulatory Activities (MedDRA^®^), which provides a standardized set of terms related to medical conditions [25]. The intensity of laboratory events was assessed using the ACTG “Division of AIDS (DAIDS)–Table for Grading the Severity of Adult and Paediatric Adverse Events–Version 2017” [26], where events graded 3 or 4 were classified as intense. In April 2023, a WHO recommendation for managing ocular toxicity was published [27], prompting the formal initiation of research into ocular manifestations associated with miltefosine use at the Reference Center for Leishmaniasis at the René Rachou Institute (*Centro de Referência em Leishmanioses do Instituto René Rachou*–CRL-IRR). These manifestations were categorized as AEs of particular interest (AESI).

### Statistical analysis

Descriptive analyses included absolute and relative frequencies for categorical events, as well as measures of central tendency and dispersion for continuous variables, depending on their distribution pattern (normal or non-normal). For variables with a normal distribution, descriptive statistics employed the mean and standard deviation, while for non-normally distributed variables, the median and interquartile range (IQ 25%–75%) were used.

The Chi-square test was employed for categorical variables in the univariate analysis, while the Pearson correlation test was used for correlation analyses. The t-test was applied to compare means between two groups, and analysis of variance (ANOVA) was utilized for comparisons of more than two groups, specifically for continuous variables with a normal distribution. For continuous variables with a non-normal distribution, non-parametric tests were used to compare the medians in independent samples. The Mann-Whitney U test was used to compare two groups, and the Kruskal-Wallis test was employed to compare three or more groups.

Multiple logistic regression was conducted using the backward technique to examine the relationship between explanatory variables and the outcomes of interest, specifically premature treatment interruption and the occurrence of clinical and laboratorial SAEs, classified as grades 3 and 4. The adequacy of the model was assessed using the Hosmer-Lemeshow test. The exposure variables that showed a moderate statistical association (p<0.20) were included in the multivariate model and adjusted through logistic regression after testing for collinearity and ensuring the independence of variables. A statistical significance level of 5% was applied to all tests.

This project was approved by the Research Ethics Committee of the Institute René Rachou/FIOCRUZ Minas (registration number 4.702.086; CAAE:45481421).

## Results

### Study participants and aspects of treatment

A total of 157 patients treated with miltefosine in the Brazilian state of Minas Gerais between July 2021 and August 2023 were eligible for this analysis. The ages of the patients ranged from 25 to 90 years, with a median age of 62 years (IQR 52.5–73.5). The median weight was 71.9 kg (IQR 84.6–63.0), and there was a predominance of males (68.8%). In terms of occupation, 61 (38.9%) patients were retired, 25 (15.9%) were farmers, and in 15 (9.6%) cases, professional activity was not reported.

Of the 157 patients, 122 had comorbidities (Table 1). Among the total patients included in this analysis, 144 (91.7%) were tested for HIV infection, and all results were negative. There were nine women (5.7%) of childbearing age, five (55.6%) of whom were using hormonal contraceptives combined with barrier methods. Three women (33.3%) had undergone salpingectomy or hysterectomy, and one (11.1%) was in sexual abstinence. Table 1 presents the main clinical and demographic characteristics of the patients.

**Table 1:** Demographic and clinical data of the study participants.

Characteristic	Number of patients (%)
**Sociodemographic data**	
**Gender**	
Male	108 (68.8)
Female	49 (31.2)
**Age**	
0–18 years	0 (0.0)
18–60 years	69 (43.9)
>60 years	88 (56.1)
**Women of childbearing age**	
Yes	9 (18.4)
No	40 (81.6)
**Educational level**	
Elementary education	39 (24.8)
High school	21 (13.4)
Higher education	20 (12.7)
Illiterate	8 (5.1)
Unknown	69 (43.9)
**Race** ^1^	
White	38 (24.2)
Non-white	99 (63.1)
Not informed	20 (12.7)
**Occupation**	
Retired	61 (38.9)
Farmer	25 (15.9)
Not informed	15 (9.6)
**Area**	
Urban and peri-urban	93 (59.2)
Rural	62 (39.5)
Unknown	2 (1.3)
**Clinical data** **Comorbidities**	
Arterial hypertension	90 (57.3)
Diabetes mellitus	27 (17.2)
Heart disease	24 (15.3)
Kidney disease	11 (7.0)
Liver disease	1 (0.6)
Immunosuppression	1 (0.6)
HIV infection^2^	0 (0.0)
Others ^3^	41 (26.1)
**Clinical Form**	
Cutaneous (less than three lesions)	77 (49.0)
Cutaneous (more than three lesions)	35 (22.3)
Mucosal	45 (28.7)
**Diagnostic tests**	
PCR^4^	67 (42.7)
Parasitological microscopic identification	52 (33.1)
Histopathological (presence of amastigote form)	36 (22.9)
Cultural	2 (1.3)
**Type of clinical entry**	
New cases	93 (59.2)
Relapses	64 (40.8)

^1^ In Brazil, stratification in health services is defined by self-reporting, based on the classification categories as established by the Brazilian Institute of Geography and Statistics (Instituto Brasileiro de Geografia e Estatística—IBGE), which includes white, black, brown/mixed, asian, and Indigenous.

^2^144 patients were tested for HIV infection.

^3^Endocrine-metabolic, neuropsychiatric, respiratory, gastrointestinal, rheumatologic, other infectious, cardiovascular, and oncological diseases.

^4^ PCR: polymerase chain reaction. Source: developed by the authors.

The *Leishmania* species were not identified; however, it is historically known that *Leishmania braziliensis* is the most prevalent species in the region [22]. Miltefosine was chosen after other medications failed to show a response (42%) due to restrictions on alternative treatments (40.8%), for convenience (14%), and because of hypersensitivity to other therapies (3.2%). A completed treatment—defined as administration for over 90% of the planned duration—was achieved in 138 patients (87.9%), with the duration of medication use ranging from 27 to 71 days and a median of 29 days (IQR 28–30). Treatment was discontinued for 19 patients; of these, 12 cases (63.2%) were due to a medical decision resulting from increased creatinine levels, two patients (10.5%) lost to follow-up, one patient (5.2%) discontinued due to an AE reported as Chikungunya infection requiring hospitalization, and a justification was not reported in four cases (21%).

### Adverse events in patients treated with miltefosine

A total of 317 AEs were identified among 121 patients (77.1% of cases), averaging two events per patient treated with miltefosine (Table 2). Of these, 230 were classified as clinical adverse abnormalities, with a predominance of gastrointestinal-related events (n = 174; 76%), followed by musculoskeletal and connective tissue disorders. Notably, arthralgia was reported in 22 patients (14%). Among the 147 patients with available laboratory monitoring results, 68 (46.3%) exhibited at least one altered parameter during follow-up. Laboratory AEs accounted for 27.5% of the total AEs recorded (n = 87), most of which were related to the renal and urinary systems, particularly elevations in creatinine, which occurred in 49 episodes (56.3% of laboratory AEs). Hospitalization during miltefosine use was reported in six patients (3.8%), indicating an SAE. These cases included three female and three male patients, aged between 62 and 86 years, with five (83.3%) of them having pre-existing comorbidities. The diagnoses at the hospital discharge were acute renal failure, acute myocardial infarction, erosive esophagitis, Chikungunya, urinary tract infection, and cholelithiasis.

**Table 2:** Adverse events reported during miltefosine treatment.

Adverse events distributed by organ-system	Number of affected patients (%)
**Gastrointestinal**	
Nausea	79 (50.3)
Vomiting	41 (26.1)
Diarrhea	25 (15.9)
Abdominal pain	24 (15.3)
**Musculoskeletal**	
Arthralgia	22 (14.0)
Myalgia	3 (1.9)
**Cutaneous**	
Rash	1 (0.6)
**General**	
Fever	1 (0.6)
**Ocular**	5 (3.2)
**Laboratory abnormalities**	
Elevation in creatinine	49 (33.3)
Elevation in TGP	11 (7.5)
Elevation in TGO	10 (6.8)
Elevation in bilirubins	4 (2.7)
Elevation in amylase	3 (2.0)
Elevation in lipase	3 (2.0)

TGP, glutamic-pyruvic transaminase; TGO, glutamic-oxalacetic transaminase.

From April 2023, approximately 18 months after the initiation of this study, active research on ocular manifestations during miltefosine use formally commenced. Ocular issues were reported by five patients (3.2%), with complaints including visual changes (blurred vision or reduced visual acuity) and three cases of ocular irritation. One patient presented with a CL lesion on the face, accompanied by contiguous palpebral involvement. During miltefosine treatment, this patient experienced increased ocular irritation and secretion, resulting in conjunctivitis, which progressively improved throughout the course of CL treatment; however, no additional tests were performed. All patients with ocular complaints were referred for ophthalmological evaluation, and in each case, no new specific ocular conditions were confirmed. One patient with reduced visual acuity was diagnosed with presbyopia and prescribed corrective lenses. In all cases, the ocular manifestation was mild and intermittent during the treatment phase. Except for the patient with refractive conditions, symptoms were resolved without the need for specific treatment.

Models for multiple analyses were employed to explore factors associated with the following outcomes of interest: the occurrence of laboratory AEs, the occurrence of laboratory SAEs, and treatment discontinuation. Given the clinical relevance of the findings, the outcomes of severe creatinine elevation (grades 3 and 4) during miltefosine treatment and hospitalization (an SAE) were also evaluated (Table 3). No high correlations (above 0.7) were identified between the independent variables in the collinearity analysis among the variables.

**Table 3:** Factors significantly associated with outcomes of interest in multiple analysis modeling.

Outcome of interest	Associated factors (variables in the model)	P-value	Exp (B)	95% CI
Occurrence of creatinine elevation*	Systemic arterial hypertension	0.007	3.772	1.450–9.815
	Age	0.029	1.032	1.003–1.062
	Mucosal clinical form	0.021	3.030	1.183–7.761
Occurrence of creatinine elevation grade 3 or 4	Elevated pre-treatment creatinine levels	0.008	30.667	2.422–388.276
Hospitalization	Age	0.019	1.142	1.022–1.276
Incomplete treatment	Elevated pre-treatment creatinine levels	0.039	4.763	1.080–21.002
	Age	0.000	1.173	1.076–1.276

Exp (B), beta exponent (magnitude of association); 95% CI, 95% confidence interval.

* Elevation above reference values or change in grade in patients with a baseline above the reference values.

The most frequently observed laboratory alteration was elevation in serum creatinine levels, occurring in 33.3% of cases. The multiple analysis confirmed that this elevation was significantly associated with “hypertension,” “age,” and the “mucosal form of leishmaniasis.” Regarding the intensity of laboratory AEs, seven patients (4.76%) experienced elevations in serum creatinine levels, reaching grade 3 or 4. Notably, four (57.1%) of these seven patients already had serum creatinine levels above the upper limit of normality before starting miltefosine treatment, a condition linked to the occurrence of grade 3 or 4 increases in creatinine. All AEs related to liver or pancreatic functions were graded as 1 or 2.

The early treatment discontinuation rate was 11.8%, with 63.2% of these cases linked to increased creatinine levels. Factors significantly associated with treatment discontinuation included age and pre-existing renal dysfunction. No instances of pregnancy were recorded among women treated with miltefosine or among women whose male partners were treated with miltefosine during the observation period.

Considering temporality, consistency with current knowledge, plausibility, and the presence of other potential causes for the SAEs, four out of six events were classified as unrelated. These patients had diagnoses of acute myocardial infarction, cholelithiasis, urinary infection, and arboviral infection. Therefore, in this series, the rate of patients with SAEs deemed related to miltefosine treatment was 1.3% (2/157). Additionally, three out of five patients with ocular AEs were classified as possibly related to the treatment due to temporal association, even though no specific ocular condition was confirmed.

## Discussion

Safety data from clinical trials in the field of neglected diseases remain extremely limited. Despite the potential to track many exposed patients, current surveillance data often relies on spontaneous reports, lacking both prospective follow-up and systematic methods, which restricts its contributions. This underscores the need for new pharmacovigilance (PV) approaches with greater scientific rigor to assess the safety profile of medications more accurately [28]. These methods are crucial for determining the quantitative aspects of medicine safety, identifying specific risk factors and high-risk groups, and providing valid clinical characteristics of issues associated with specific drugs. Public health programs, which typically manage large populations in an organized and structured manner, present excellent platforms for capturing quantitative PV data [29]. By systematically recording the number of patients treated, drugs administered, and doses given, these programs enable more precise monitoring and evaluation of drug safety. Implementing improved PV systems within these frameworks is vital for enhancing the safety and efficacy of treatments for neglected diseases.

Few studies have focused on the safety profile of miltefosine for CL. The present study provides valuable real-world evidence, offering insights into how the drug performs in a specific population under real-world conditions, where patients often take multiple medications and have underlying health conditions. As expected, most clinical events related to miltefosine involved gastrointestinal manifestations, followed by musculoskeletal complaints. The most frequent laboratory abnormality was elevation in serum creatinine levels, which was severe in some cases, followed by increases in transaminases. This pattern of changes in biochemical tests differs from that observed in visceral leishmaniasis cases but has been previously reported among oncology patients [30, 31]. The rise in creatinine levels was associated with factors such as “hypertension,” “age,” and the “mucosal form of leishmaniasis.” Severe creatinine elevations (grades 3 and 4) were significantly linked to patients who had renal dysfunction before starting miltefosine treatment. Similarly, “hospitalization” was associated with “age,” while “incomplete treatment” was significantly related to both “age” and “elevated pre-treatment creatinine levels.” These findings highlight the connection between renal toxicity and pre-existing kidney dysfunction, emphasizing the impact of these factors on the use of miltefosine, particularly among older adults.

In this pilot implementation study, most patients were over 60 years old, differing from the expected average of CL patients in Brazil, who are predominantly young adults [32]. The preference for miltefosine among older adults likely stems from the restriction of antimony use in those over 50 years old, making it a more viable option for this age group. The age group of 60 and over is potentially the one that would benefit from miltefosine treatment, which is particularly relevant given the aging population and the well-established correlation between drug toxicity and age population [33, 34]. Our findings confirm that most reported AEs with miltefosine are associated with the gastrointestinal system, such as nausea and vomiting. These effects have been consistently demonstrated, including the initial studies with miltefosine in cancer patients [35] and other clinical trials in leishmaniasis [14–16]. The second most frequently affected system was the musculoskeletal and connective tissue, manifested as arthralgia. Although the mechanism remains unclear, at least one study has reported gout episodes in three patients treated with miltefosine, all of whom had a previous history of the disease but without accompanied by hyperuricemia [36]. In our series, serum uric acid levels were not measured, and no cases of arthritis were reported. Additionally, none of the patients with arthralgia required treatment discontinuation. According to miltefosine’s manufacturer instructions filed with the FDA, treatment interruption due to arthritis is only noted for visceral leishmaniasis [37]. Although our data do not corroborate a link between miltefosine and the risk of gouty arthritis, the evidence collected thus far suggests that investigating hyperuricemia in patients with joint complaints who are using miltefosine is warranted, particularly in those with a prior history of gout.

Among the laboratory changes, elevated creatinine was the most frequently observed abnormality, an event described in the product’s leaflet as occurring in approximately 10% of patients but reported three times more frequently in this study. Only 7% of the 157 cases had a confirmed diagnosis of nephropathy or baseline creatinine levels above reference values. However, with around 57% of patients having hypertension and 17% having diabetes, a higher percentage of patients likely had pre-existing renal dysfunction that went undiagnosed before miltefosine treatment. Pre-treatment dysfunction was linked to more severe creatinine elevations. These findings suggest the need for more frequent monitoring of renal function, particularly in patients with chronic cardiovascular conditions related to the risk of renal deterioration, such as hypertension and diabetes, as well as in older adult patients and those with the mucosal form of CL.

After miltefosine’s registration, new safety information regarding reproductive and ocular AEs emerged. The drug’s effect on spermatogenesis [38] prompted a review of its FDA registration in May 2021 [37]. A few years later, multiple cases of ocular events in patients undergoing extended treatment with miltefosine for post-kala-azar dermal leishmaniasis (PKDL) in India [39, 40] led WHO to issue an alert in 2023 [27]. In our study, three patients presented ocular complaints possibly related to miltefosine. However, no correlation between the treatment and changes in the ophthalmological examination could be identified. The current evidence remains insufficient to rule out a causal link, highlighting the importance of post-marketing clinical trials as vital tools for vigilance [41]. This study focuses on a single intervention, limiting the ability to directly compare the toxicity profile with other available therapeutic options. Furthermore, the toxicity profile of antimonial derivatives, which remain the most commonly used treatment for CL, has not been thoroughly evaluated, as studies specifically designed for safety monitoring are lacking [42]. Antimonial treatments for leishmaniasis, including meglumine antimoniate, are associated with significant toxicity, particularly affecting the liver, pancreas, and heart. Hepatotoxicity, characterized by elevated liver enzymes (e.g., AST and ALT), affects approximately 30–70% of patients. Pancreatitis, indicated by elevated serum amylase and lipase levels, has been reported in 25–75% of cases, depending on the patient population and dosage [43, 44]. Cardiotoxicity, including electrocardiogram (ECG) changes such as prolonged QT intervals, occurs in approximately 5–20% of patients. This condition can lead to rare but severe complications, including arrhythmias and sudden cardiac events. Although there have been reports of sudden death, pancreatitis, and fatal arrhythmias associated with treatment, their exact incidence remains poorly defined [45]. However, it is important to note that the parenteral use of antimonials is restricted in patients with organ dysfunction and is limited for those over 50 years of age—factors that are not considered contraindications for miltefosine. This limitation complicates a comparative analysis of the two treatments. The population treated in the miltefosine implementation study was older and had more comorbidities than those typically treated with antimonials. In contrast, antimony intralesional infiltration, while primarily associated with local events, typically causes mild and transient reactions at the administration site, posing a very low risk of SAEs [46]. Despite the high number of AEs associated with miltefosine, most were mild to moderate, with SAEs linked to age and pre-existing conditions.

The miltefosine discontinuation rate was low—approximately 12%; however, this figure may be underestimated due to the challenges associated with validating information from secondary sources [13]. Age and elevated serum creatinine levels prior to the start of miltefosine treatment were factors associated with early treatment interruption, suggesting a potential link to pre-existing renal function. Regarding treatment adherence, 50% of patients required more than 28 days to complete at least 90% of the planned doses, probably due to temporary interruptions caused by toxicity or logistical challenges. Although shorter treatment periods with miltefosine [47] and cases of early treatment interruptions [36] have not reduced efficacy in some studies, the impact of multiple treatment interruptions on effectiveness remains unknown.

One of the main challenges in implementing miltefosine within health systems is its teratogenicity, which requires special care from both prescribers and patients. Reproductive toxicity mandates that patients of childbearing age avoid pregnancy not only during treatment but also for three months following exposure to miltefosine. In the study group, nine women (5.7%) were of childbearing age, and no pregnancies were recorded during this pilot phase. This relatively low percentage of women of reproductive age in this sample, which does not represent the expected disease distribution by sex and age, may reflect the exclusion of this group due to difficulties in adhering to contraception requirements, concerns about compliance with these measures, or resistance from women to using contraception. Adherence to contraceptive methods and monthly pregnancy monitoring could not be assessed in this study; however, these are fundamental aspects that necessitate recording and monitoring tools.

The high percentage of cases lacking general laboratory monitoring underscores the challenges in ensuring regular follow-up and conducting necessary tests, particularly for patients in remote areas. These challenges emphasize the urgent need for a detailed and specific approach to managing the teratogenic risks associated with miltefosine. Strengthening partnerships with local health systems is essential to establish basic infrastructure, including access to pregnancy testing and contraceptive methods. Therefore, the presence of women of childbearing age may be the main barrier to the large-scale implementation of miltefosine. Mitigating this risk could lead to resistance from patients and professionals, a concern that should be assessed in acceptability studies.

This study has several limitations. The reliance on secondary data sources, which were contingent on the voluntary submission of monitoring forms filled out by various professionals lacking specific training and in-depth knowledge of PV, limits the generalizability of the present results. Additionally, some barriers stem from the lack of adherence of prescribing physicians to complete the monitoring forms, highlighting the need to enhance health record systems. Furthermore, only laboratory events could be classified in terms of the intensity of AEs. The likely underreporting of events, assumed by the difference between the rate of events observed in the CRL-IRR compared to other services in Minas Gerais, suggests that the patient safety centers, although mandatory, were inactive or had a sub-optimal performance in most units [22]. Conversely, we included a significantly large number of patients, representing the heterogeneous population characteristic of real-world drug use.

This study collected safety data for a population often excluded from clinical trials, thereby enhancing our understanding of potential toxicity in specific subgroups. These findings suggest that miltefosine’s safety profile varies based on the treated disease. Specifically, we identified an association between laboratory AEs and mucosal involvement in CL, possibly associated with a worse health condition of those patients, generally older and with comorbidities. These results may have practical implications for the clinical management of CL in Brazil and PV strategies. The continuous evaluation of the miltefosine implementation process, along with real-world monitoring of AEs, is essential in pursuing more effective and safer treatments for leishmaniasis in Brazil. These efforts align with the process of health technology assessments within the Brazilian Public Healthcare System, which should be seen as complementary and inseparable actions. Further studies are needed to either validate or refute these results, as they are currently based on hypotheses rather than assertive conclusions.

Real-world studies provide valuable information on drug-drug interactions and the impact of miltefosine when used alongside other diseases, offering a more comprehensive understanding of its safety profile. Post-marketing surveillance has identified gastrointestinal disturbances, hepatotoxicity, and nephrotoxicity as notable adverse effects of miltefosine, effects that were less pronounced in clinical trials [48].

Public health initiatives must thoroughly assess and detail the risks medications pose to individuals and communities. This is essential for reducing harm, promoting the appropriate use of medicines, maintaining public trust in these initiatives, and monitoring issues arising from medication errors and substandard drugs. Therefore, new approaches are required to track the quantitative aspects of medication safety, which will help identify risk factors and high-risk groups more effectively and provide detailed descriptions of AE linked to specific medicines in targeted populations. This PV strategy model holds potential for application to new technologies in other neglected disease control programs.

## Supporting information

S1 Table Notifiable Diseases Information System (SINAN) form. (DOCX)

S2 Table Miltefosine request form. (DOCX)

S3 Table Safety monitoring forms. (DOCX)
