# Supplementary material for: Safety profile of miltefosine in the treatment of cutaneous leishmaniasis
Source: PLoS One. 2024 Dec 13;19(12):e0315710. doi: 10.1371/journal.pone.0315710 (PMC11643273; doi:10.1371/journal.pone.0315710)
Supplement: S1 Table — (DOCX) [file pone.0315710.s001.docx]

**Federative Republic of Brazil Ministry of Health**

**Nº**

## SINAN

INFORMATION SYSTEM FOR NOTIFIABLE DISEASES

CASE INVESTIGATION FORM **AMERICAN TEGUMENTARY LEISHMANIASIS**

#### CONFIRMED CASE:

**Cutaneous Leishmaniasis:** any individual with the presence of a cutaneous ulcer with a granular base and infiltrated edges, confirmed by laboratory diagnosis or clinical epidemiology.

**Mucosal Leishmaniosis:** any individual with the presence of an ulcer in the nasal mucosa with or without perforation or loss of the nasal septum, potentially affecting lips and mouth (palate and nasopharynx), confirmed by laboratory diagnosis or clinical epidemiology.

**Notification Type**

**General Information**

**1**

**2** Disease

2 - Individual

Código (CID10)

**3** Date of Notification

**AMERICAN TEGUMENTARY LEISHMANIASIS**

| **|** |

**|** | | |

**4** FU

# |

**5** Municipality of Notification Code (IBGE)

# | | | | |

1. Health Unit (or other notifying source)

Code

1. Date of Diagnosis

# | | | | | |

| **|** | **|** | | |

1. Patient’s Name
2. Date of Birth

| **|** | **|** | | |

**Individual Notification**

1. Age

### | |

**14** Educational level

1. - Hour
2. - Day
3. -Month
4. - Year

**11** Gender M - Male

F - Female

I - Ignored

1. Pregnant

*1-1st Trimester 2-2st Trimester 3-3st Trimester*

*4- Ignored Gestational Age 5-Not 6- Not Aplicable 9-Ignored*

1. Rece/Color

*1-White 2-Black 3-Yellow*

*4-Brown 5-Indigenous 9- Ignoreted*

0- Illiterate 1-1st to 4th grade incomplete (old primary or 1st grade) 2- 4th grade complete (old primary or 1st grade)

3-5th to 8th grade incomplete (old gymnasium or 1st grade) 4- Complete elementary education (old gymnasium or 1st grade) 5- Incomplete high school (old high school or 2nd grade) 6- Complete high school (old high school or 2nd grade) 7- Incomplete higher education 8- Complete higher education 9- Ignored 10- Not applicable

- 1. SUS Card Number
  2. Mother’s name

# | | | | | | | | | | | | | | |

- 1. FU

# |

- 1. **Municipality of Residence**

Code (IBGE)

# | | | | |

- 1. District
  2. Street

**Residence Data**

**22** Number

**21** Neighborhood

**23** Apartment number, house...

1. Other

Code

# | | | | |

1. Other
2. Landmark
3. ZIP CODE

# | | | | - | |

1. Telephone Number

# | | | | | | | | |

1. Zone 1 - Urban 2 - Rural

3 - Periurnan 9 - Ignored

1. Country (if resident outside Brazil)
2. Date of investigation

**Antec. Epidem.**

## Additional Case Information

1. Occupation

| **|** | **|**

**|**

# | | |

1. Presence of Lesion 1 - Yes 2 - No

**Clinical Date**

Cutaneous

Mucosal

1. In Case of Mucosal Lesion, Are There Cutaneous Scars

1 - Yes 2 - No

1. HIV Co-infection

1 - Yes 2 - No

9 - Ignored

**41** Treatment Start Date

| **Lab Data** |
| --- |
| **Case Clas.** |
| **Treatment** |

**37** IRM

1 - Positive 2 - Negative 3 - Not Performed

**36** Direct Parasitology

1 - Positive 2 - Negative 3 - Not Performed

**39** Classification

1 – New Case 2 - Relapse 3-Transfer 9- Ignored

**40** Clinical Form

1 - Cutaneous 2 - Mucosal 9- Ignored

**42** initial Drug Administered

**38** Histopathology

1 - Parasite visualized 2 - Compatible

3 – Not Compatible 4 – Not Performed

| **|** | **|** | | |

1 – Pentavalent Antimonial 2 – Anphotericin B 3 - Pentamidine 4 - Others 5 – Not Used

**43** Weight

| | Kg

**44** Prescribed Dose in mg/kg/day Sb+5

1 - Less than 10 **2** - Greater or equal to 10 and less than 15 **3** - equal to 15

**4** - Greater than 15 and less than 20 **5** - Greater or equal to 20

**45** Total dosage **46**

| | Vials

Other Drug prescribed in case of Treatment Failure

1 - Amphotericin B 2 - Pentamidine 3 - Others 4 - Not Applicable

American Cutaneous LeishmaniasisSinan NET

SVS 27/09/2005

**47** Confirmation Criteria

**48** Epidemiological Classification Classification

1 - Laboratory

2 - Clinical-Epidemiological EEEEEmidemiological

- Autochthonous AAAAAAAAAutoAutochthonous Autochthonous

2 - Imported 3 - Undetermined

#### Likely source of infection

**49** Is the case autochthonous of the residence municipality? 1-Yes 2-No 3-Undetermined

**50** FU **51**Country

|

**52** Município

Código (IBGE)

**53** District

**54** Address

| | | | |

**55** Is the disease related to work? 1 - Yes 2 – No 9 - Ignored

**56** Case Evolution

1-Cure

2-Discontinuation 3-Death

4-Death by other causes 5-Transfer 6-Change of diagnosis

**57** Date of Death **58** Closing Date

|

**|**

|

**|**

| | |

|

**|**

|

**|**

| | |

**Additional Information and Observations**

**Conclusion**

Travel (dates and places visited in the six months before the onset of signs and symptoms)

| Date | State | City | Contry |
| --- | --- | --- | --- |
|  |  |  |  |
|  |  |  |  |
|  |  |  |  |

Extra important information

**Professional Data**

Name

Signature

American Cutaneous Leishmaniasis

Municipality/Health Center

Health Center Number

| | | | | |

Sinan NET

SVS 27/09/2005
