# Supplementary material for: Safety profile of miltefosine in the treatment of cutaneous leishmaniasis
Source: PLoS One. 2024 Dec 13;19(12):e0315710. doi: 10.1371/journal.pone.0315710 (PMC11643273; doi:10.1371/journal.pone.0315710)
Supplement: S2 Table — (DOCX) [file pone.0315710.s002.docx]

|  | | | | | | | | | | | | | | | | | | | | | | | | | | | | | | | | | | | | | | |
| --- | --- | --- | --- | --- | --- | --- | --- | --- | --- | --- | --- | --- | --- | --- | --- | --- | --- | --- | --- | --- | --- | --- | --- | --- | --- | --- | --- | --- | --- | --- | --- | --- | --- | --- | --- | --- | --- | --- |
| Miltefosine Request | | | | | | | | | | | | | | | | | | | | | | | | | | | | | | | | | | | | | | |
| **Prescriber’s information** | | | | | | | | | | | | | | | | | | | | | | | | | | | | | | | | | | | | | | |
| **Name:** | |  | | | | | | | | | | | | | | | | **Specialty:** | |  | | | | | | | | | | | | | | | | | |  |
| **Medical registration number:** | |  | | | | | | | | | | | | | | | | **Email:** | |  | | | | | | | | | | | | | | | | | |  |
| **Telephone number:** | |  | | | | | | | | | | | | | | | | | | | | | | | | | | | | | | | | | | | |  |
| **Requesting health center information:** | | | | | | | | | | | | | | | | | | | | | | | | | | | | | | | | | | | | | | |
| **Name:** | |  | | | | | | | | | | | | | | | | **Telefone:** | |  | | | | | | | | | | | | | | | | | |  |
| **Address/City/State:** | |  | | | | | | | | | | | | | | | | | | | | | | | | | | | | | | | | | | | |  |
| **Patient information** | | | | | | | | | | | | | | | | | | | | | | | | | | | | | | | | | | | | | | |
| **Name:** | |  | | | | | | | | | | | | | | **Date of birth** | | | |  | | | | | | | | | | **Age** | |  | | | | | |  |
| **Mother’s name:** | |  | | | | | | | | | | | | | | **Telephone number** | | | |  | | | | | | | | | | | | | | | | | |  |
| **ID:** | |  | | | | | | | | | | | | | | **Address/City/State** | | | |  | | | | | | | | | | | | | | | | | |  |
| **Gender:** | |  | | **male** | | | |  | | **female** | | | | | | **Women of childbearing age?:** | | | | **Yes** | | | |  | **No** | | |  | | |  | | | | |  |  |  |
| **Diagnostic tests information:** | | | | | | | | | | | | | | | | | | | | | | | | | | | | | | | | | | | | | | |
| **Parasitological microscopic identification** | | **(  ) positive (  ) negative (  ) inconclusive (  ) not carried out**    **(  ) positive (  ) negative (  ) inconclusive (  ) not carried out**  **(  ) positive (  ) negative (  ) inconclusive (  ) not carried out** | | | | | | | | | | | | | | | | | | | | | | | | | | | | **Date:** | |  | | | | | |  |
| **Molecular (PCR)** | |  |  |  |  |  |  |  |  |  |  |  |  |  |  |  |  |  |  |  |  |  |  |  |  |  |  |  |  |  |  |  | | | | | |  |
| **Histopathological (presence of amastigote form)** | |  |  |  |  |  |  |  |  |  |  |  |  |  |  |  |  |  |  |  |  |  |  |  |  |  |  |  |  |  |  |  | | | | | |  |
| **Other:** | |  | | | | | | | | | | | | | | | | | | | | | | | | | | | |  |  |  | | | | | |  |
| **Clinical information** | | | | | | | | | | | | | | | | | | | | | | | | | | | | | | | | | | | | | | |
| **FORM CLINICAL FORM** | |  | | **CL≤ 3 lesions** | | | | | | | | |  | | | **CL3-6 lesions** | | | | | | | | | | | | | | | | | | | | | |  |
|  |  |  | | **CL> 6 lesions** | | | | | | | | |  | | | **cutaneous and mucosal** | | | | | | | | | | | | | | | | | | | | | |  |
|  |  |  | | **ML** | | | | | | | | | | | | | | | | | | | | | | | | | | | | | | | | | |  |
| **COMORBIDITY** | |  | | **YES** | | | |  | | **NO** | | | | | | | | | | | | | | | | | | | | | | | | | | | |  |
|  |  |  | | Arterial hypertension | | | |  | | **Mellitus Diabetes** | | |  | | | **Kidney disease** | | | | | | | |  | **heart disease** | | | | | | | | | | |  |  |  |
|  |  |  | | **Liver disease** | | | | | | | | |  | | | **Immunosuppression:** | | | | | | | | | | | | | | | | | | | | | |  |
|  |  | **Other:** | |  | | | | | | | | | | | | | | | | | | | | | | | | | | | | | | | | | |  |
| **Prescription choice justification (Check one or more alternatives):** | | | | | | | | | | | | | | | | | | | | | | | | | | | | | | | | | | | | | | |
|  | **First-line (restriction to other therapeutic alternatives)** | | | | | | | | | | |  | | | **Lack of response from other medications** | | | | | | | | | | | | | | | | | | | | | |  |  |
|  | **First-line (convenience)** | | | | | | | | | | |  | | | **Lack of response from miltefosine** | | | | | | | | | | | | | | | | | | | | | |  |  |
|  | **Treatment continuity (2nd cycle)** | | | | | | | | | | |  | | | **hypersensitivity to the administration of other treatments** | | | | | | | | | | | | | | | | | | | | | |  |  |
| ***Other. Specify.** |  | | | | | | | | | | | | | | | | | | | | | | | | | | | | | | | | | | | |  |  |
| ***Previous treatments:** |  | | **No previous treatment** | | | | | | | | *** Etiological agent:** | | |  | | | **Leishmania braziliensis** | | | | | | | | | | | | | | | | | |  |  |  |  |
|  |  | | **parenteral meglumine antimoniate** | | | | | | | |  |  |  |  | | | **Leishmania guyanensis** | | | | | | | | | | | | | | | | | |  |  |  |  |
|  |  | | **Intralesional parenteral meglumine antimoniate** | | | | | | | |  |  |  |  | | | **Leishmania amanzonensis** | | | | | | | | | | | | | | | | | |  |  |  |  |
|  |  | | **Liposomal Amphotericin B** | | | | | | | |  |  |  |  | | | **OTHER:** | | | | | | | | | | | | | | | | | |  |  |  |  |
|  |  | | **Amphotericin B** | | | | | | | |  |  |  |  | | | **Unspecified** | | | | | | | | | | | | | | | | | |  |  |  |  |
|  |  | | **Pentamidine isethionate** | | | | | | | | **OBS.** | | |  | | | | | | | | | | | | | | | | | | | | |  |  |  |  |
|  |  | | **Pentoxifylline associated with meglumine antimoniate** | | | | | | | |  |  |  |  |  |  |  |  |  |  |  |  |  |  |  |  |  |  |  |  |  |  |  |  |  |  |  |  |
|  |  | | **Miltefosine** | | | | | | | |  |  |  |  |  |  |  |  |  |  |  |  |  |  |  |  |  |  |  |  |  |  |  |  |  |  |  |  |
|  |  | | *** OTHER(DESCRIBE):** | | | | | | | |  |  |  |  |  |  |  |  |  |  |  |  |  |  |  |  |  |  |  |  |  |  |  |  |  |  |  |  |
| **PRESCRIPTION INFORMATION** | | | | | | | | | | | | | | | | | | | | | | | | | | | | | | | | | | | | | | |
| ***Prescription date (1^st^ Cycle):** | |  | | | | | | | | | | | | | | | | | | | | | | | | | | | | | | | | | | | |  |
| ***Weight(kg):** | ***Dosage** | | | | | | ***Daily dosages** | | | | | ***Treatment duration** | | | | | | | | | | | ***Amount requested (number of capsules)** | | | | **Obs:** | | | | | | |  |  |  |  |  |
|  |  | | **2,5mg/kg/day** | | |  | | | **8/8 hours** | |  | | | **14 days** | | | | | | | |  | | | |  | | | | | | |  |  |  |  |  |  |
|  |  | | ***Other:** | | |  | | | **12/12 hours** | |  | | | **Other:** | | | | | | | |  |  |  |  |  |  |  |  |  |  |  |  |  |  |  |  |  |
|  | ***Justify, other:** | | | | | | | | | | | *** Justify, other:** | | | | | | | | | | | | | | | | |  | | | | | | | |  |  |
| **INFORMATION OF THE MILTEFOSINE 50MG CAPSULE DISPENSARY UNIT** | | | | | | | | | | | | | | | | | | | | | | | | | | | | | | | | | | | | | | |
| ***Unit Name(SIGAF):** | | | | |  | | | | | | | | | | | | | | **CNES:** | |  | | | | | | | | | | | | | | | | | |
| ***Business Registration Number:** | | | | |  | | | | | | | | | | | | | | ***E-mail:** | |  | | | | | | | | | | | | | | | | | |
| **Unit Address** | | | | |  | | | | | | | | | | | | | | ***Pharmacist Registration Number:** | |  | | | | | | | | | | | | | | | | | |
| ***Telephone number** | | | | |  | | | | | | | | | | | | | |  | | **Check if the unit holds a health permit** | | | | | | | | | | | | | | | | | |
| *** Pharmacist’s Name (SIGAF)** | | | | |  | | | | | | | | | | | | | |  | | **Check if the unit holds a valid technical regularity certificate** | | | | | | | | | | | | | | | | | |
|  | | | | | | | | | | | | | | | | | | | | | | | | | | | | | | | | | | | | | | |
|  | | | | | | | | | | | | | | | | | | | | | | | | | | | | | | | | | | | | | | |
| **Signature** | | | | | | | | | | | | | | | | | | | | | | | | | | | | | | | | | | | | | | |
|  | | | | | | | | | | | | | | | | | | | | | | | | | | | | | | | | | | | | | | |
