# Supplementary material for: Safety profile of miltefosine in the treatment of cutaneous leishmaniasis
Source: PLoS One. 2024 Dec 13;19(12):e0315710. doi: 10.1371/journal.pone.0315710 (PMC11643273; doi:10.1371/journal.pone.0315710)
Supplement: S3 Table — (DOCX) [file pone.0315710.s003.docx]

| **Miltefosine Safety Monitoring Form** |
| --- |

| **Name:** \|_ \|_ \|_ \|\|_ \|_ \|_ \|\|_ \|_ \|_ \|\|_ \|_ \|_ \|\|_ \|_ \|_ \|\|_ \|_ \|_ \|\|_ \|_ \|_ \|    **Date of birth**\|_ \|_ \|_ \|\|_ \|_ \|_ \| **Gender** \|_ \|female \|_ \| male  **Mother’s name:** \|_ \|_ \|_ \|\|_ \|_ \|_ \|\|_ \|_ \|_ \|\|_ \|_ \|_ \|\|_ \|_ \|_ \|\|_ \|_ \|_ \| |
| --- |

| **Information about the treatment** |
| --- |
| **Dosage** ( ) 50 mg every 8 hours ( ) 50 mg every 12 hours  **Starting date ________   End date _________**  **Early discontinuation** (  ) Yes   (  ) No  **Discontinuation cause** (  ) adverse effects (  ) pregnancy (  ) patient’s choice |

| **Clinical information** |
| --- |
| **Clinical form:** (  )  CL ≦ 3 lesions   (   ) CL 3-6 lesions  (  ) >6 lesions                         (  ) cutaneous-mucosal    (   ) ML |
| **Comorbidities** (   ) Yes  (   ) No  (  ) Arterial hypertension  (  ) Diabetes Mellitus ( ) Other _________________  (  ) Kidney disease (  ) Heart disease  (  ) Liver disease (  ) Immunosuppression |
| **Contraceptive Method: ______________________ + _____________________________** |

| **Clinical and Laboratory Evaluation** | | | | | |
| --- | --- | --- | --- | --- | --- |
| **Test** | **Baseline** | **7th-10th day** | **15th-18th day** | **21st-24th day** | **Extra __/__/__** |
| Urea | _______ mg/dL | _______ mg/dL | _______ mg/dL | _______ mg/dL | _______ mg/dL |
| Creatinine | _________mg/dL | _________mg/dL | _________mg/dL | _________mg/dL | _________mg/dL |
| Bilirrubins | _________ mg/dL | _________ mg/dL | _________ mg/dL | _________ mg/dL | _________ mg/dL |
| TGO | _________U/L | _________U/L | _________U/L | _________U/L | _________U/L |
| TGP | _________ U/L | _________ U/L | _________ U/L | _________ U/L | _________ U/L |
| Amylase | __________U/L | __________U/L | __________U/L | __________U/L | __________U/L |
| Lipase | __________U/L | __________U/L | __________U/L | __________U/L | __________U/L |
|  | | | | | |
| Nausea | |  |  |  |  |
| Vomiting | |  |  |  |  |
| Abdominal pain | |  |  |  |  |
| Diarrhea | |  |  |  |  |
| Rash | |  |  |  |  |
| Fever | |  |  |  |  |
| Others: _____________________ | |  |  |  |  |
| ______________________ | |  |  |  |  |
| ______________________ | |  |  |  |  |
